# Non‐Contrast Assessment of Blood–Brain Barrier Permeability to Water: Improved Signal Modeling and Data Acquisition

**DOI:** 10.1002/mrm.70168

**Published:** 2025-11-02

**Authors:** Wen Shi, Jiani Wu, Yifan Gou, Jie Song, Zhiyi Hu, Zihan Wang, Dengrong Jiang, Rebecca Veenhuis, Leah Rubin, Yulin Ge, Zixuan Lin, Hanzhang Lu

**Affiliations:** ^1^ Department of Biomedical Engineering Johns Hopkins University School of Medicine Baltimore Maryland USA; ^2^ The Russell H. Morgan Department of Radiology & Radiological Science Johns Hopkins University School of Medicine Baltimore Maryland USA; ^3^ Department of Neurology Johns Hopkins University School of Medicine Baltimore Maryland USA; ^4^ Department of Radiology New York University Grossman School of Medicine New York New York USA; ^5^ F. M. Kirby Research Center for Functional Brain Imaging Kennedy Krieger Research Institute Baltimore Maryland USA

**Keywords:** blood–brain barrier, cerebral venous system, permeability, venous transit time, WEPCAST MRI

## Abstract

**Purpose:**

Water‐extraction‐with‐phase‐contrast‐arterial‐spin‐tagging (WEPCAST) MRI is a non‐contrast method to estimate the blood–brain barrier (BBB) permeability to water. Similar to other arterial‐spin‐labeling (ASL) based techniques, signal‐to‐noise ratio is a limitation. This study aims to enhance its reliability via theoretical and experimental improvements.

**Methods:**

We propose a generalized‐venous‐signal (GVS) model to describe the signal evolution of WEPCAST MRI, with which the control and labeled signals can be utilized to simultaneously estimate the water extraction fraction (*E*) and venous transit time (VTT). We conducted studies to test its feasibility and inter‐visit reproducibility. We further made an experimental improvement by adding a 1‐min blood T_1_ scan and investigating its benefit in reducing inter‐subject variations.

**Results:**

When applying the GVS model‐based method at different locations along the superior sagittal sinus (SSS), VTT increased from anterior to posterior segments while *E* remained constant, consistent with known physiology. BBB permeability‐surface‐area‐product (PS) revealed a significantly lower CoV of 5.0% ± 4.1% when using the GVS method, in comparison with 8.9% ± 6.5% using the peak‐detection method (*p* = 0.002). Blood T_1_ was found to be 1725.7 ± 37.2 ms in males and 1799.2 ± 122.4 ms in females. After including subject‐specific blood T_1_ in the parametric estimation, inter‐subject CoV in PS was found to be 6.8%, compared with a CoV of 14.2% when using an assumed blood T_1_ (*p* = 0.004). VTT estimated from WEPCAST was consistent with that measured with a dedicated sequence (*R* = 0.757, *p* = 0.011).

**Conclusion:**

The reliability of WEPCAST MRI for the measurement of BBB permeability can be improved by incorporating GVS model and individual blood T_1_.

## 1 Introduction

Blood–brain barrier (BBB) is a tight and selectively permeable barrier that protects the brain from neurotoxins and maintains a stable microenvironment for neural activities and metabolism [1]. Breakdown of the BBB is increasingly recognized as an important hallmark in several neurological diseases, such as cognitive impairment and dementia [2, 3], multiple sclerosis [4], cerebral small vessel disease [5, 6], and neurological sequelae of COVID‐19 [7, 8, 9]. Therefore, it is desirable to develop reliable techniques to non‐invasively assess BBB integrity in a clinically feasible setting.

Compared to conventional approaches that aim to measure BBB permeability to large molecules, for example, albumin and Gadolinium‐based contrast agents [10, 11], there has been a surging interest to measure water permeability recently because (1) water is a small molecule and its permeability is potentially more sensitive to subtle BBB disruption in the early pathogenesis of disease; (2) water has an intrinsic MR signal and its permeability measurement usually does not require contrast agents or other invasive procedures. These methods have been based on arterial spin labeling (ASL) or diffusion principles, exploiting the differences in T_2_, diffusivity, or magnetization transfer between intravascular and extravascular spins [12, 13, 14, 15, 16, 17]. Water‐extraction‐with‐phase‐contrast‐arterial‐spin‐tagging (WEPCAST) MRI was one of the ASL‐based methods, which works by selectively measuring the fraction of arterially labeled water in the veins, for example in the superior sagittal sinus (SSS), thereby quantifying the global water extraction fraction (E) and BBB permeability surface area product (PS) [18]. BBB integrity measured by WEPCAST MRI has been corroborated in animal models using biotin tracers and immunostaining of tight junction proteins [19, 20, 21], suggesting that WEPCAST MRI, without the need of contrast agent administration, can be used to estimate BBB dysfunctions that are often subtle in neurodegenerative diseases.

Despite their obvious clinical significance, remaining challenges associated with all water BBB techniques are that signal‐to‐noise ratio (SNR) of the data is low and there exist certain confounding factors in the measurements. Therefore, substantial technical development/optimization work is still needed to improve the reliability of the measurement and reduce variability across healthy individuals. For the WEPCAST MRI technique, the current estimation approach, referred to as the peak‐detection method, is based on the assumption that the peak WEPCAST signal along the SSS appears in the middle of the labeled bolus, thus has a known venous bolus arrival time (BAT) [22]. While this assumption is expected to be valid on a cohort level, on an individual level, noise contamination may cause errors in the bolus timing assumption, resulting in variations across measurements and subjects. Another assumption used in the previous method was the fixed blood T_1_ value. Variations in the individual blood T_1_ will cause additional errors in the estimated BBB permeability.

Here, we propose a new WEPCAST MRI signal model, referred to as the generalized venous signal (GVS) model, which alleviates the need to assume bolus timing. Indeed, by modeling the control/label signals in WEPCAST MRI (as opposed to modeling the subtraction signal only), one can simultaneously estimate the water extraction fraction (E) and the venous transit time (VTT), which denotes the transit time for the blood to travel from the capillary to the vein, specifically SSS in the present study [23]. Next, we addressed the blood T_1_ assumption by adding a blood T_1_ measurement to the acquisition and verified the benefit of individual‐level blood T_1_ in reducing inter‐subject variations in BBB permeability measure. A total of three studies were conducted in this report.

## 2 Methods

### 2.1 WEPCAST MRI Pulse Sequence and Generalized Venous Signal Model

**FIGURE 1:**
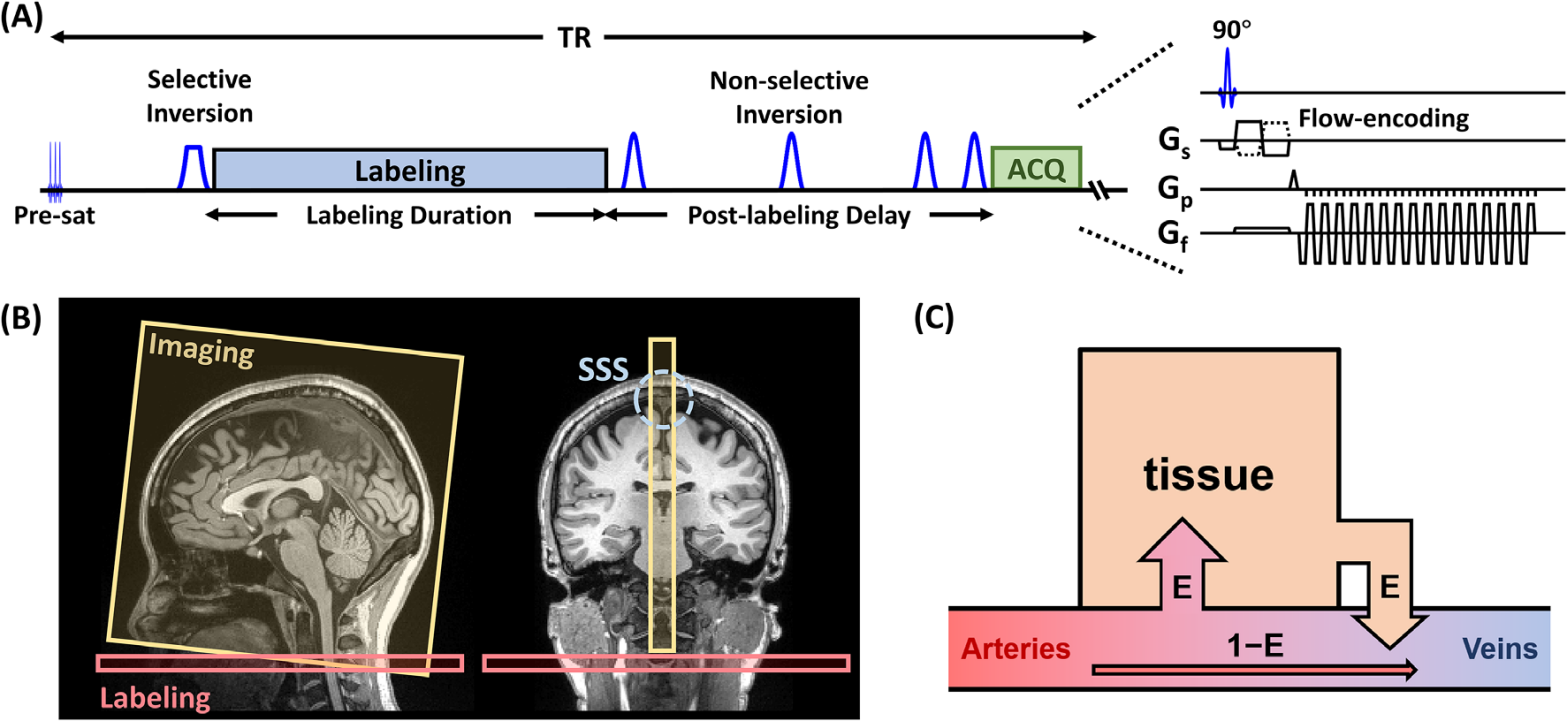
Illustration of the WEPCAST MRI technique. (A) Pulse sequence diagram of WEPCAST MRI. (B) Typical imaging position of WEPCAST MRI in sagittal and coronal views. (C) A schematic diagram showing that the water spins in the veins primarily come from the tissue quantified by an extraction fraction *E*, with the remaining unextracted spins from the arteries.

Figure 1A,B shows the pulse sequence diagram and imaging position of WEPCAST MRI, respectively. Briefly, a labeling module employing the pCASL scheme is applied at the cervical spine to invert the spins in the feeding arteries [18]. Unlike ASL that aims to measure the labeled spins in the tissue, WEPCAST MRI measures the labeled spins that remain in cerebral vessels and drain into major cerebral veins, for example, SSS. To highlight the ASL signals and avoid partial volume effects, a flow‐encoded phase‐contrast approach is employed in acquisition. Background suppression is also applied in the WEPCAST pulse sequence using hyperbolic‐secant adiabatic pulses that ensure inversion efficiency and minimize B1 sensitivity.

In this study, we propose a generalized venous signal (GVS) model to characterize the venous signals in WEPCAST MRI. Water extravasation occurs through exchange across the BBB at the capillary‐tissue interface. As shown in Figure 1C, water spins in the cerebral veins originate from two sources. The majority of the spins derive from the tissue with a fraction of E, referred to as water extraction fraction, due to the relatively high permeability of water across the BBB [24]. The other portion is the unextracted water spins from the arteries. Given this framework, the magnetization in the vein, $M_{v}$, at a time of data acquisition, t, can be written as

(1) $$M_{v}\left(t\right)=E\cdot M_{v,\text{tissue}}\left(t\right)+\left(1-E\right)\cdot M_{v,\text{artery}}\left(t\right)$$

where $M_{v,\text{tissue}}$ and $M_{v,\text{artery}}$ are the magnetization of the water spins in the veins from tissue and arteries (in units of MR signal/100 mL blood), respectively.

As described in an earlier report [23], the longitudinal relaxation of venous spins originally from the tissue follows a two‐phase process. Initially, these spins relax in the tissue compartment where the perfusion spins also influence the relaxation process. Thus, in the absence of an RF pulse, the magnetization can be written as the following equation, similar to an ASL kinetic model [25, 26]:

(2) $$\begin{array}{cc} \frac{dM_{\text{tissue}}\left(t^{\prime }\right)}{dt^{\prime }} & =\frac{M_{0,\text{tissue}}-M_{\text{tissue}}\left(t^{\prime }\right)}{T_{1,\text{tissue}}}\\ & \;+f\left(M_{\text{artery}}\left(t^{\prime }\right)-\frac{M_{\text{tissue}}\left(t^{\prime }\right)}{\lambda }\right),\;0\leq t^{\prime }<t-\delta_{v} \end{array}$$

where $M_{\text{tissue}}$ is the tissue magnetization (in units of MR signal/100 g tissue) and $M_{0,\text{tissue}}$ is the equilibrium magnetization of tissue. $M_{\text{artery}}$ is the magnetization of arterial blood (in units of MR signal/100 mL blood) following the blood T_1_ relaxation. $T_{1,\text{tissue}}$ represents the tissue T_1_. f denotes the cerebral blood flow (CBF). λ is the blood–brain partition coefficient. $\delta_{v}$ represents the VTT. t is the time of data acquisition.

It should be pointed out that background suppression RF pulses have been employed in the pulse sequence (Figure 1A). Thus, they will alter the magnetizations described above. However, these effects are equivalent to breaking down the period in Equation (2) into several piecewise periods, with the magnetizations inverted between adjacent periods. These effects can be easily accounted for in Bloch simulations.

Once these spins are exchanged into the blood at the time of $t-\delta_{v}$, the magnetization is written in the following to account for the blood–brain partition coefficient:

(3) $$\begin{array}{c} M_{v,\text{tissue}}\left(t^{\prime }\right)=\frac{M_{\text{tissue}}\left(t^{\prime }\right)}{\lambda },\;t^{\prime }=t-\delta_{v} \end{array}$$

Then these spins follow the blood T_1_ relaxation, and the associated Bloch equation can be written as.

(4) $$\begin{array}{c} \frac{dM_{v,\text{tissue}}\left(t^{\prime }\right)}{{\mathrm{dt}}^{\prime }}=\frac{M_{0,v}-M_{v,\text{tissue}}\left(t^{\prime }\right)}{T_{1,b}},\;t-\delta_{v}<t^{\prime }\leq t \end{array}$$

where $M_{0,v}$ is the equilibrium magnetization of venous blood (in units of MR signal/100 mL blood). $T_{1,b}$ denotes the blood T_1_. The background suppression RF pulses can be accounted for again by changing Equation (4) to piecewise expressions.

For the $M_{v,\text{artery}}$ term in Equation (1), these spins always stay in the vessels and the magnetization recovers with $T_{1,b}$ relaxation. Let $\delta_{a}$ and $\delta_{v}$ be the arterial transit time (ATT) and VTT, respectively. $M_{v,\text{artery}}$ thus follows.

(5) $$\begin{array}{c} \frac{dM_{v,\text{artery}}\left(t^{\prime }\right)}{{\mathrm{dt}}^{\prime }}=\frac{M_{0,v}-M_{v,\text{artery}}\left(t^{\prime }\right)}{T_{1,b}},\;t-\left(\delta_{a}+\delta_{v}\right)\leq t^{\prime }\leq t \end{array}$$

Background suppression RF pulses can be accounted for by piecewise expressions.

Note that Equations (1–5) are applicable for both label and control conditions, with the only difference being the expression of $M_{v,\text{artery}}$. In the label condition, $M_{v,\text{artery}}$ was inverted. In the control condition, $M_{v,\text{artery}}$ was not inverted.

In WEPCAST MRI, the tissue spins will reach the vein after $\delta_{v}$ seconds. In reality, considering the relatively long time of $\delta_{v}$ (> 3000 ms), there is an expected dispersion in $\delta_{v}$. Here, we assumed that $\delta_{v}$ follows a normal distribution $p∼N\left(\delta_{v},\sigma^{2}\right)$, and the standard deviation σ was assumed to be 1161.7 ms based on a previous report [23]. As a result, Equation (1) is expanded to:

(6) $$\begin{array}{cc} M_{v}\left(t\right) & =E\cdot \int p\left(\delta_{v}\right)M_{v,\text{tissue}}\left(t,\delta_{v}\right)d\delta_{v}\\ & \;+\left(1-E\right)\cdot \int p\left(\delta_{v}\right)M_{v,\text{artery}}\left(t,\delta_{v}\right)d\delta_{v} \end{array}$$

In this study, $\delta_{a}$ was assumed to be proportional to $\;\delta_{v}$. Note that here $\delta_{a}$ denotes the time it takes for the water molecules to travel from the labeling site to the tissue‐capillary junction. It is slightly different from the transit time obtained from standard multi‐delay ASL, which denotes the time it takes for labeled water to reach the small arteries in the imaging voxel [27]. Previous studies have shown that $\delta_{a}$ was 1901 ms [28], and $\delta_{v}$ was 3423 ms (based on Shi et al. with adjustment of updated $T_{1,b}$) [23]. Thus, a fixed ratio of 0.555 between $\delta_{a}$ and $\delta_{v}$ was assumed in our data fitting.

### 2.2 Simulations to Generate a Dictionary for Parametric Estimation

In the GVS framework described above, there are two unknown parameters, the water extraction fraction (E) and venous transit time ($\delta_{v}$). Using WEPCAST MRI, we obtain two experimental measures of $M_{v}$, one under the control condition and the other under label. Thus, we have a simple problem of two measures and two unknowns. We can therefore generate a dictionary using simulations to map between unknowns and experimental measures. Specifically, Equations (2–6) were used for the simulation, in which $T_{1,b}$ (female/male) = 1742/1667 ms [29], $T_{1,t}$ = 1056 ms [30], λ = 0.9 mL/g [31], labeling efficiency = 0.86 [32]. $p\left(\delta_{v}\right)$ is represented by a discrete histogram with 300 bins evenly spaced from 0 to 9000 ms. f is based on the CBF measurement from the experiment. Because f is different for each participant, a dictionary is generated for each participant.

**FIGURE 2:**
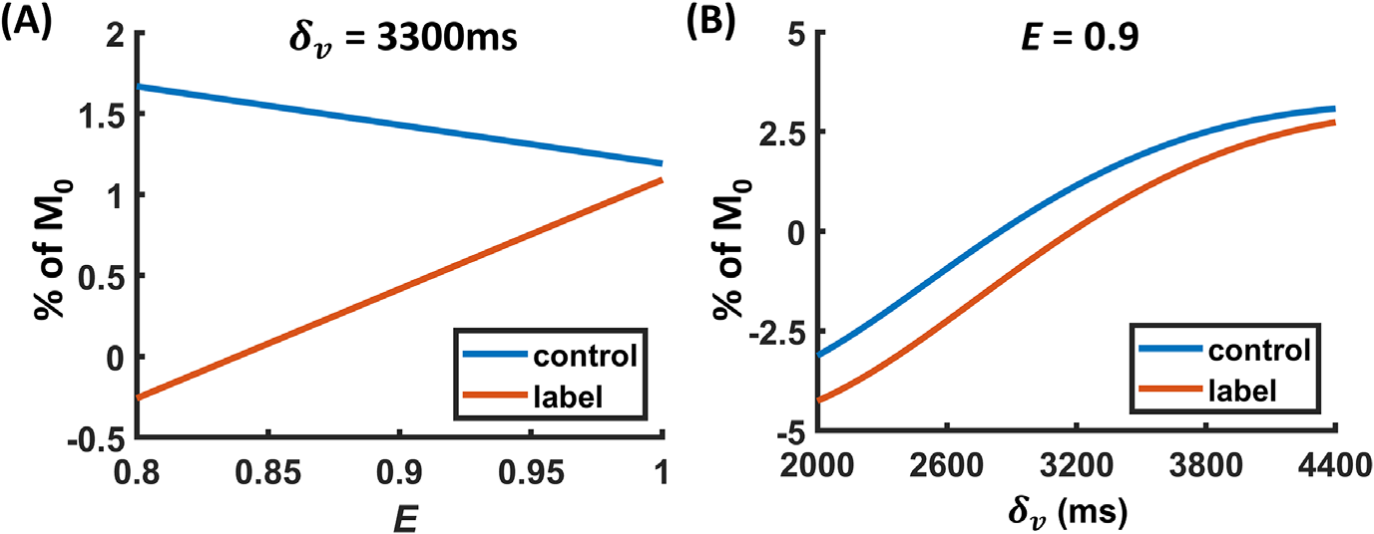
Numerical simulation of venous signals in WEPCAST MRI based on the generalized venous signal model. (A) Simulated control and label WEPCAST signals as a function of *E* with a $\delta_{v}$ of 3300 ms and (B) as a function of $\delta_{v}$ with an *E* of 0.9. A cerebral blood flow of 55 mL/100 g/min was assumed in the simulation.

Figure 2A,B shows how control and labeled venous signal changes when E and $\delta_{v}$ alter. It can be observed that E and $\delta_{v}$ have distinctive effects on control and labeled signals, allowing them to be easily teased apart in parametric estimation. *f* was fixed at 55 mL/100 g/min in this illustration.

**FIGURE 3:**
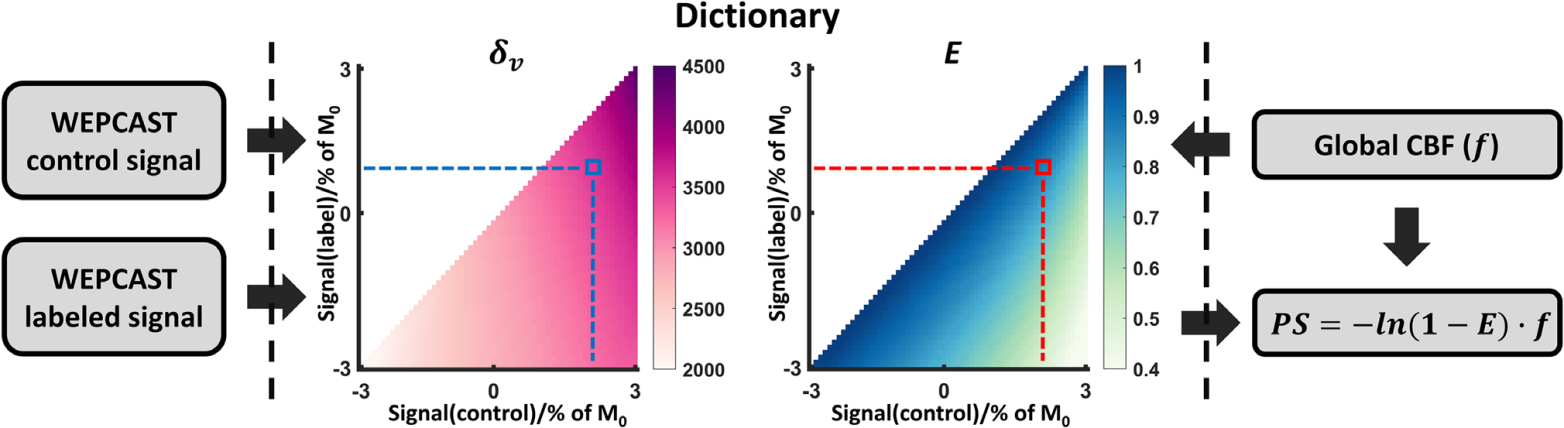
A schematic diagram explaining the steps used in GVS data processing. Global CBF (f) is quantified by phase‐contrast MRI. A dictionary of water extraction fraction (E) and venous transit time ($\delta_{v}$) is then generated using the specific f, providing a one‐to‐one correspondence between the experimental WEPCAST control/labeled signals and the estimated parameters. E and $\delta_{v}$ are determined from the best‐match entry in the dictionary. PS is derived from *E* and f according to the Renkin‐Crone model.

Figure 3 shows a schematic diagram of the GVS method to estimate the BBB permeability. First, phase‐contrast quantitative flow data were used to estimate f. Next, based on the particular f, a dictionary was generated using simulations that provide a one‐to‐one correspondence between the experimental measures and the estimated parameters. The dictionary sampled E from 0.4 to 0.999 with a step size of 0.001 and $\delta_{v}$ from 2000 ms to 4500 ms with a step size of 25 ms. The dictionary generation takes approximately 22 s on an Intel Core I9‐11900 processor. Then, WEPCAST control and labeled signals as well as the corresponding M_0,*v*_ were obtained from the SSS. Using the dictionary, the E and $\delta_{v}$ were estimated. Finally, using E and f, the BBB permeability‐surface area product (PS) was computed using the Renkin‐Crone equation [33, 34]:

(7) $$\begin{array}{c} \mathrm{PS}=-\ln\left(1-E\right)\cdot f \end{array}$$

### 2.3 MRI Experiments

We conducted three experimental studies in this report. All studies were approved by the Johns Hopkins University Institutional Review Board and performed on a Siemens 3 T Prisma scanner (Siemens Healthcare, Erlangen, Germany) using a 32‐channel head coil. A total of 26 healthy subjects (25 ± 4 years, 12 males and 14 females) were enrolled. Informed written consent was obtained before participation.

WEPCAST MRI was performed in the mid‐sagittal plane that contains the entire SSS with the following parameters unless otherwise specified: single slice, single‐shot EPI readout, TR/TE = 9200/9.5 ms, labeling duration (τ) = 4 s, post‐labeling delay (PLD) = 3 s, FOV = 200 × 200 × 10 mm^3^, voxel size = 3.1 × 3.1 × 10 mm^3^, number of control/label pairs = 10, flow‐encoding direction = FH, $V_{\mathrm{enc}}$ = 20 cm/s, GRAPPA = 3, scan duration = 6.9 min. Note that, although a thick slice was used, the flow‐encoded phase‐contrast acquisition will allow the measurement of venous signal only. The PLD value was chosen so that the center of the labeled bolus was approximately located at the posterior SSS. Individual variations in blood travel time will make it slightly off‐center. However, with the relatively long labeling duration (4 s used in the protocol), the WEPCAST signal will still be close to maximum. An $M_{0}$ scan was performed for signal normalization using an identical phase‐contrast scheme except for TR = 10 s. PC MRI was conducted at four arteries (left/right internal carotid arteries and left/right vertebral arteries) to measure the total cerebral blood flow [32, 35], with the following imaging protocol: TR/TE = 16/10.2 ms, FOV = 200 × 200 × 5 mm^3^, voxel size = 0.5 × 0.5 × 5 mm^3^, $V_{\mathrm{enc}}$ = 40 cm/s, scan duration = 13 s. A T_1_‐MPRAGE scan was also performed and whole brain volume was obtained using the Computational Anatomy Toolbox (CAT) [36]. Global CBF, f, can then be quantified as the total blood flow divided by the brain volume. The image processing and analysis were performed using in‐house MATLAB (MathWorks, Natick, MA, USA) scripts.

#### 2.3.1 Study I: Implementation of GVS‐WEPCAST MRI and Spatial Distribution of Physiological Parameters

We first implemented the technique and characterized the spatial distribution of the measured parameters $\delta_{v}$ and E along the anterior–posterior direction of the SSS. This is to partially validate the techniques based on known physiology. Since venous blood travels from anterior to posterior direction in the SSS, one expects that $\delta_{v}$ values will be lower in the anterior end and gradually increase in the downstream posterior segments. On the other hand, *E* is not expected to show major change across a healthy brain [37, 38], thus, its anterior–posterior profile is supposed to be flat.

Eight healthy subjects (26 ± 6 years, 3 males and 5 females) were enrolled. In this study, we conducted WEPCAST MRI with an anterior‐to‐posterior flow‐encoding to better image the spatial profile across the SSS. Eight non‐overlapping ROIs were manually delineated on the SSS along the anterior–posterior direction. $\delta_{v}$ and E were estimated at each ROI using the GVS‐based framework described above. To study the regional dependence of $\delta_{v}$ and E, a linear mixed‐effect model was employed with the location along the SSS as a fixed effect and subject as a random‐intercept effect.

#### 2.3.2 Study II: Inter‐Visit Test–Retest Reproducibility and Comparison With Existing Method

Next, we sought to investigate the test–retest reproducibility of the technique. Because physiological MRI measures tend to exhibit a greater degree of day‐to‐day variations (compared to anatomic and structural MRI, for example), we chose to examine the inter‐visit reproducibility, as opposed to intra‐session and inter‐session variability examined previously [22]. Ten healthy volunteers (25 ± 2 years, 5 males and 5 females) were enrolled. Each participant underwent WEPCAST, PC, and T_1_‐MPRAGE scans on two different days (interval between scans = 24 ± 18 days). No subjects reported changes in health status between the two scan sessions.

Additionally, we compared results from the proposed GVS‐based processing approach with those using the existing peak‐detection approach. The details of the peak‐detection method can be found in a previous report [22]. Briefly, in the peak‐detection method, the WEPCAST signal along the SSS was determined and the location of the peak signal was identified. This location was then assumed to correspond to the middle of the labeling bolus, with a BAT of half of the labeling duration plus the post‐labeling delay (τ/2 + PLD). E can then be calculated from the peak signal.

Pearson correlation was calculated to assess the consistency between the two processing methods. We also compared the two approaches in terms of their coefficients of variation (CoV) using a paired t‐test. Bland–Altman plot was also used to evaluate the reproducibility.

We also conducted sensitivity tests to investigate the dependence of *E*, PS, and $\delta_{v}$ on GVS model parameters. Specifically, the estimation was performed again on the same experimental data when altering each model parameter by ±10%, including the standard deviation of VTT distribution (σ), ATT ($\delta_{a}$) and $T_{1,t}$.

#### 2.3.3 Study III: Further Improving the Reliability of GVS‐WEPCAST MRI by the Measurement of Individual Blood T_1_

Studies I and II assumed a fixed blood T_1_ based on the sex of the participants. However, blood T_1_ is known to have considerable inter‐subject variations, which could propagate to errors in the PS quantification [22]. Therefore, in this study, we examined whether additional measurement of blood T_1_ on an individual basis can further reduce the variations in PS across healthy volunteers.

**FIGURE 4:**
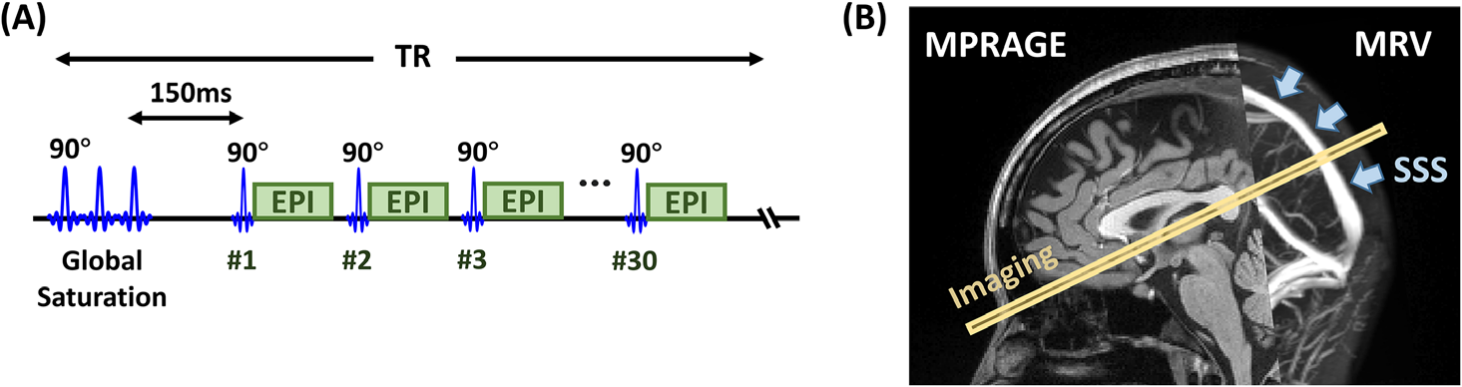
Pulse sequence used for the blood T_1_ measurement. (A) MRI pulse sequence diagram. The sequence starts with a global saturation to remove spin history, followed by a series of EPI acquisitions at an interval of 150 ms. A total of 30 images were acquired per TR. (B) Typical position of the imaging slice. The imaging plane was placed to intersect the splenium of the corpus callosum and the anterior commissure, resulting in the slice being approximately perpendicular to the posterior SSS (blue arrow).

Twelve healthy subjects were enrolled (26 ± 4 years, 5 males and 7 females). WEPCAST MRI was performed with the same parameters as in study II. A Look‐Locker saturation recovery sequence was employed to measure the blood T_1_, similar to the method used in Qin et al. [39], Varela et al. [40], Wu et al. [41], and Zhang et al. [42]. Figure 4 shows the pulse sequence diagram and imaging plane position of the blood T_1_ sequence. The single‐slice imaging plane intersected the splenium of the corpus callosum and the anterior commissure, which makes it approximately perpendicular to the posterior SSS. The sequence started with a global saturation followed by Look‐Locker EPI‐readouts with 90° flip angles, exploiting the fact that the excited blood will be fully replenished before the next RF pulse [39, 40, 42]. The saturation recovery curve was sampled at an interval of 150 ms, with a total of 30 measurements per TR. Other imaging parameters were: TR/TE = 4570/19 ms, FOV = 200 × 200 × 3 mm^3^, voxel size = 2 × 2 × 3 mm^3^, gap between global saturation and the first excitation = 150 ms, average = 10, GRAPPA = 2, scan duration = 59 s. $T_{1,b}$ was then estimated by fitting the MR signal $S_{v}$ in the ROI of SSS according to a saturation recovery model with three coefficients [43]:

(8) $$\begin{array}{c} S_{v}\left(t\right)=S_{t}+S_{0}\cdot \left(1-e^{\frac{t}{T_{1,b}}}\right) \end{array}$$

where $S_{t}$ accounts for the residual tissue signals in the ROI due to the partial volume effect. $S_{0}$ is the equilibrium blood signal.

To further verify the validity of $\delta_{v}$ measured by WEPCAST MRI, we performed a Venous‐transit‐time‐Imaging‐by‐Changes‐in‐T1‐Relaxation (VICTR) MRI sequence on 10 out of the 12 participants, which measures $\delta_{v}$ at the posterior SSS [23]. The VICTR scan used the following imaging parameters: 15 TRs each including 8 Look‐Locker EPI‐readouts, TE = 14 ms, FOV = 150 × 150 × 10 mm^3^, voxel size = 2.3 × 2.3 × 10 mm^3^, $V_{\mathrm{enc}}$ = 35 cm/s, GRAPPA = 2, scan time = 3.9 min. The measured blood T_1_ was used in the VICTR processing.

Inter‐subject CoV was calculated for the PS from the GVS‐based results with and without individual blood T_1_ measurement. One‐tailed non‐parametric bootstrap was performed to test the CoV difference between the two sets of results, in which PS from the two methods was resampled 100 000 times to estimate the distribution of the CoV differences between results. Pearson correlation was calculated to compare $\delta_{v}$ from WEPCAST MRI and from VICTR MRI.

## 3 Results

### 3.1 Study I: Implementation of GVS‐WEPCAST MRI and Spatial Distribution of Physiological Parameters

**FIGURE 5:**
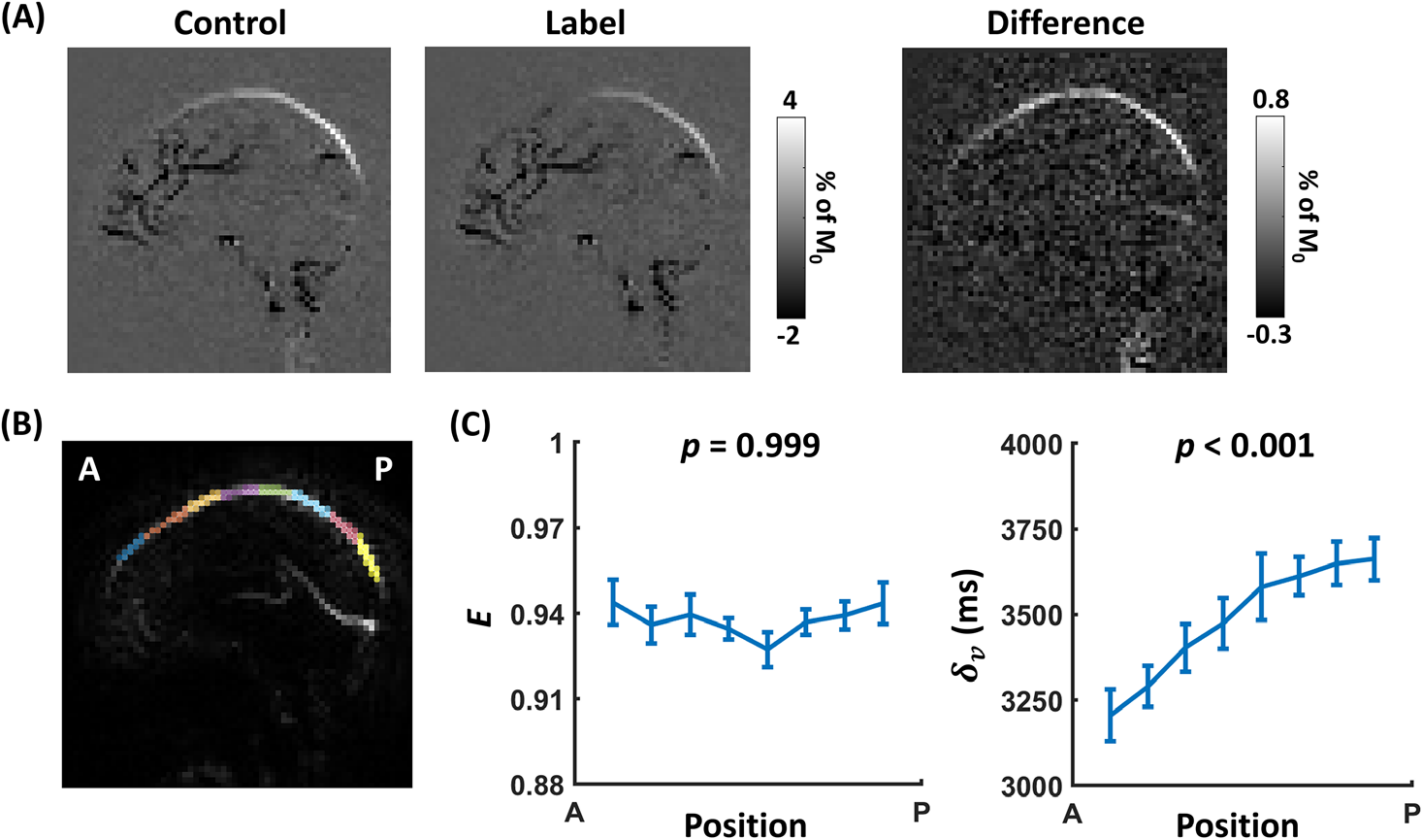
Experimental results of water extraction fraction (E) and venous transit time ($\delta_{v}$) in different segments along the SSS. (A) WEPCAST control, labeled, and difference CD images from a representative subject. (B) Eight ROIs delineated along the SSS from the same subject. (C) E and (D) $\delta_{v}$ estimates from the anterior to the posterior SSS using the proposed GVS method. Error bars denote the standard errors across subjects.

Figure 5A shows a representative WEPCAST dataset from one subject, including control, labeled, and difference CD images. It can be seen that, due to the PC flow‐encoding in the acquisition, only flowing blood can be seen in the control and labeled CD images. ASL signals in the SSS can be clearly observed in the difference CD image. Figure 5B shows 8 ROIs drawn along the anterior‐to‐posterior direction on SSS.

Figure 5C shows the results of E and $\delta_{v}$ from the GVS model. Linear mixed‐effect model analysis revealed that E does not change across different segments of the SSS (*β* = −1.488 × 10^−6^, *p* = 0.999), suggesting a stable water exchange across brain regions for healthy individuals. In contrast, $\delta_{v}$ increased from anterior to posterior SSS (*β* = 68.2, *p* < 0.001), in agreement with the flow direction of venous blood in the SSS.

### 3.2 Study II: Inter‐Visit Test–Retest Reproducibility and Comparison With Existing Method

**FIGURE 6:**
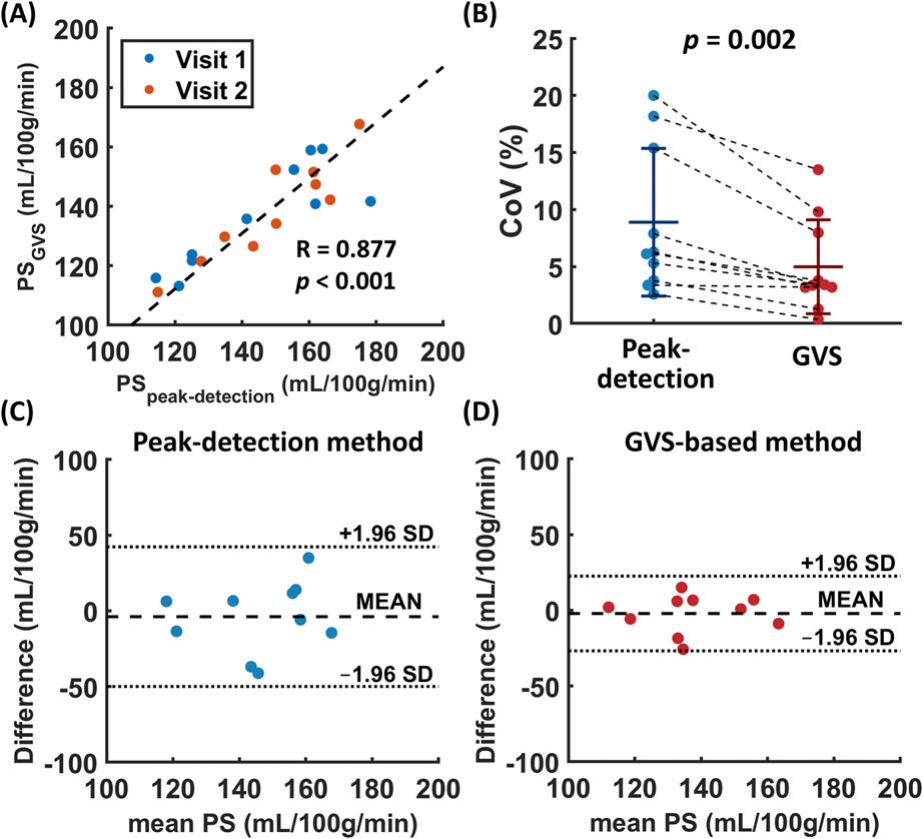
Comparison of WEPCAST results between peak‐detection and GVS method. (A) Scatter plot of PS values obtained from peak‐detection and GVS‐based approaches. The dashed line represents the linear regression. (B) Beeswarm plot of inter‐visit CoV in PS for peak‐detection and GVS‐based approaches. (C) Bland–Altman plot between the PS measurements from two visits using the peak‐detection method. (D) Bland–Altman plot between the PS measurements from two visits using the GVS‐based method. GVS, generalized venous signal; PS, permeability surface area product; CoV, coefficient of variation.

Figure 6A shows the scatter plot between the PS derived from the GVS method and the peak‐detection method. An excellent consistency can be found between the two methods (*R* = 0.877, *p* < 0.001). Figure 6B shows a bee‐swarm plot of the inter‐visit CoV using the two methods. It can be seen that the GVS‐based method has a significantly lower CoV of 5.0% ± 4.1% in PS compared to 8.9% ± 6.5% from the peak‐detection method (*t* = −4.171, *p* = 0.002), indicating better inter‐visit reproducibility. Figure 6C,D shows the Bland–Altman plot of PS from the two approaches, suggesting that the GVS‐based method has a lower inter‐visit variability in PS.

Table 1 summarizes the dependency of *E*, PS and $\delta_{v}$ estimates on the GVS model assumption. As can be seen, the effects of variations in assumed parameters are relatively minor. For a 10% variation in any of the assumed parameters, the change in estimated PS is less than 5%.

**TABLE 1:** Dependence of *E*, PS and *δ*_*v*_ estimates on GVS model parameters.

Model parameter	*E* (%)	PS (mL/100 g/min)	*δ*_*v*_ (ms)
Current	90.8 ± 4.7	137.4 ± 16.7	3628.8 ± 122.5
*σ* + 10%	89.7 ± 5.2	131.0 ± 17.1	3757.5 ± 143.3
*σ* – 10%	91.7 ± 4.2	142.6 ± 15.8	3507.5 ± 106.1
*δ*_*a*_ + 10%	89.7 ± 5.0	130.7 ± 16.3	3616.3 ± 125.2
*δ*_*a*_ – 10%	91.7 ± 4.4	143.2 ± 17.6	3645.0 ± 119.4
*T*_1,*t*_ + 10%	91.5 ± 4.4	141.7 ± 17.0	3552.5 ± 140.9
*T*_1,*t*_ – 10%	90.0 ± 4.9	131.8 ± 15.8	3708.8 ± 111.0

*Note: E*, extraction fraction. PS, permeability surface area product. $\delta_{v}$, venous transit time. GVS, generalized venous signal. σ, standard deviation of VTT distribution. $\delta_{a}$, arterial transit time. $T_{1,t}$, tissue T_1_.

### 3.3 Study III: Further Improving the Reliability of GVS‐WEPCAST MRI by the Measurement of Individual Blood T_1_

**FIGURE 7:**
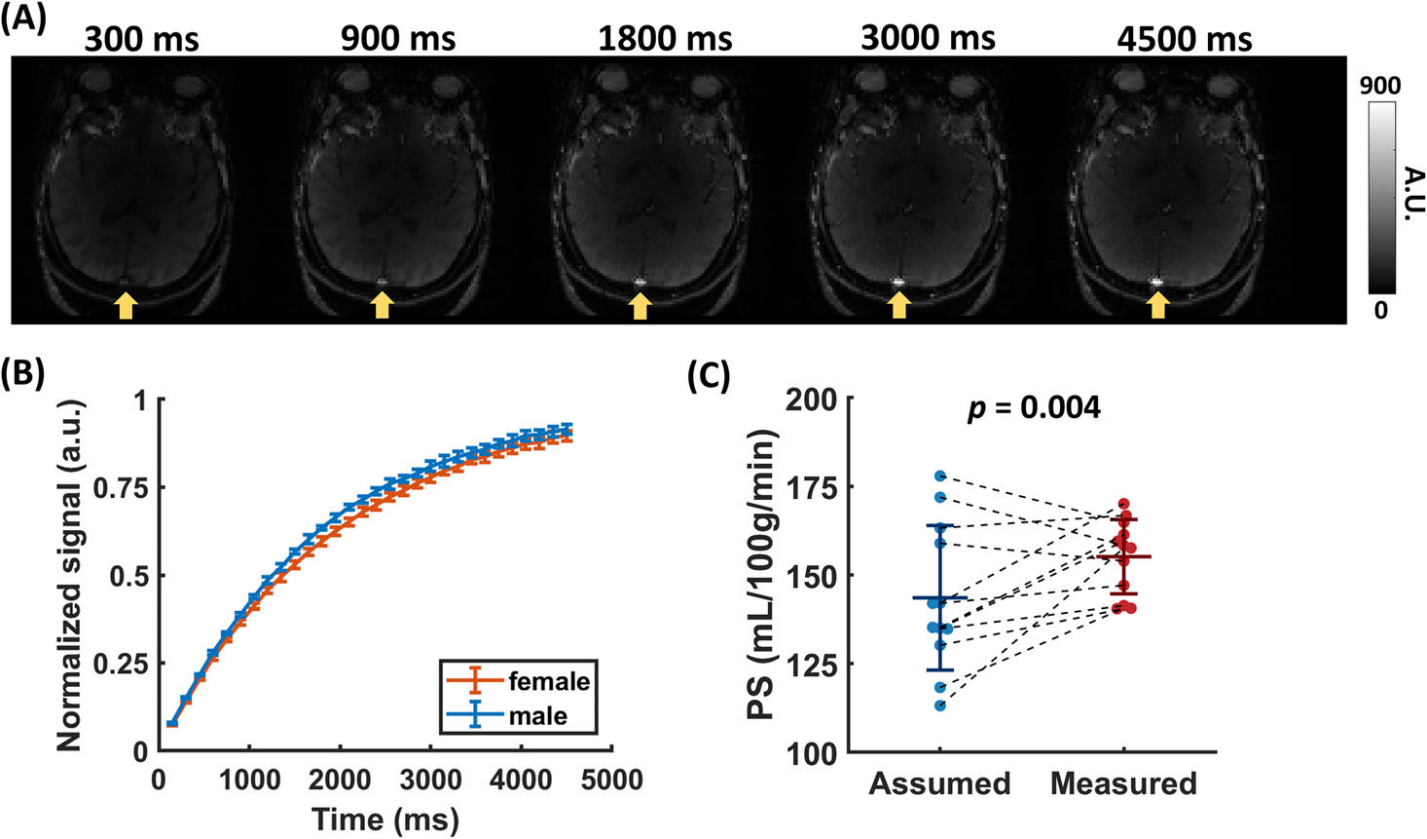
Experimental results demonstrating the benefits of blood T_1_ measurement in BBB permeability quantification. (A) Representative images from the saturation recovery sequence at a delay time of 300, 900, 1800, 3000, and 4500 ms. Signals in the SSS (arrows) can be seen to recover over time. (B) Signal as a function of time from one representative female (orange) and male participant (blue) at the posterior SSS. The signal time course was fitted to a three‐coefficient model with least squares. Error bars denote the standard error across dynamics. (C) Beeswarm plot of PS measurements with assumed and measured blood T_1_. *p*‐value was obtained from a non‐parametric bootstrap test in group CoV. PS, permeability‐surface area product.

Figure 7A shows experimental images using the blood T_1_ sequence. Signal recovery in the SSS can be clearly seen. Figure 7B shows the quantitative SSS signals as a function of recovery time from a representative male and female subject. The measured blood T_1_ was 1725.7 ± 37.2 ms for males and 1799.2 ± 122.4 ms for females (*p* = 0.228). Figure 7C compares the PS estimated from the GVS model with the assumed and individually measured blood T_1_. We found that measuring individual blood T_1_ significantly reduces the inter‐subject CoV in PS from 14.2% to 6.8% (*p* = 0.004).

**FIGURE 8:**
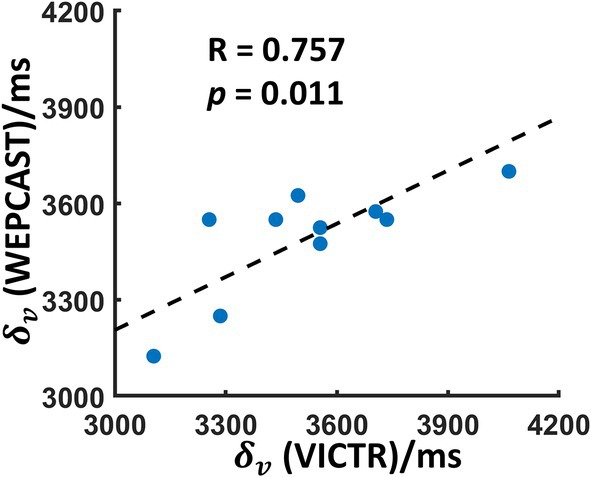
Scatter plot of venous transit time ($\delta_{v}$) obtained from VICTR MRI and WEPCAST MRI. The dashed line represents the linear regression. VICTR, venous‐transit‐time‐imaging‐by‐changes‐in‐T1‐relaxation.

Figure 8 exhibits that $\delta_{v}$ from the GVS‐based WEPCAST data has a significant correlation with $\delta_{v}$ from the VICTR MRI (*R* = 0.757, *p* = 0.011), further supporting the validity of the GVS model.

## 4 Discussion

In this work, we proposed theoretical and experimental improvements in WEPCAST MRI for more reliable measurement of BBB water permeability. A generalized venous signal model was developed to exploit both the control and labeled signals in WEPCAST MRI, instead of using the difference signal only, for simultaneous estimation of BBB permeability and VTT. This new processing method was found to reduce the inter‐visit variability of PS by 44%. Moreover, we found that an additional measurement of blood T_1_ can reduce the inter‐subject variability of PS by 52%. Collectively, these observations suggest that GVS‐based WEPCAST processing in combination with an extra blood T_1_ scan of 1 min can substantially improve the reliability of BBB permeability assessment.

A number of non‐contrast MRI techniques have been proposed to measure BBB permeability to water [12, 13, 14, 17]. WEPCAST MRI is one of them and works by measuring the arterially labeled blood signals in the vein. This technique has been tested in several animal model studies in which increases in BBB water permeability were associated with a disruption of endothelial cell tight junction and a leakage of Dextran dye [19, 44]. The inter‐visit CoV observed in the present study (5.0%) is comparable to the diffusion‐based BBB method (5.3%) [45], and slightly lower than the multi‐TE based BBB method (8.4%) [46]. For the spatial distribution of the water extraction fraction, the present study found that *E* is relatively constant across the anterior‐to‐posterior axis in healthy volunteers. This finding is consistent with the reports by Shao et al. and Zaharchuk et al. who showed that the water extraction fraction is homogenous across human and rat brain, respectively [37, 38].

WEPCAST MRI signal intensity is dependent on both the water extraction fraction, *E*, and VTT, $\delta_{v}$. A major task in quantification is to tease apart the effects of these two parameters. The original approach in WEPCAST processing was based on the assumption that the center of the labeled bolus is located within the SSS and that the center signal should be the highest due to bolus dispersion effect [22]. Therefore, by identifying the peak signal location along the SSS, one can estimate E with a known venous BAT of *τ* + PLD/2 (5 s in the current protocol). However, recent studies suggested that there exist variations in venous BAT. It was estimated that venous BAT ranges from 4.5 s to 5.6 s [23]. For a typical flow velocity in the SSS of about 12.5 cm/s and a typical length of the posterior SSS of around 10 cm [47, 48], the time it takes for the bolus peak to pass through the SSS is approximately 0.8 s. Therefore, it is likely that for subjects with very slow or very fast flow, the center of the bolus may not have arrived or already passed the SSS. This may contribute to the variations in BBB PS estimation. In the approach proposed in the present work, we exploited the fact that the control (and labeled) signal intensity contains venous BAT information. Because tissue and blood T_1_ are different, variations in VTT and consequently venous BAT will result in characteristic signal intensity in WEPCAST MRI. Based on this principle and by deriving a generalized venous signal model, we developed a theoretical framework that relates two experimental measurements (control and labeled signals) to two unknowns (*E* and $\delta_{v}$). We first tested this model in a region‐dependency study, in which we showed that $\delta_{v}$ increased from anterior to posterior segment of the SSS, in line with the known flow direction in this vessel. The measured $\delta_{v}$ also showed excellent correlation with $\delta_{v}$ measured from a dedicated sequence, VICTR MRI, although both techniques are relatively new and there still exist some differences between their quantitative values.

Compared to the previous WEPCAST processing method [22], the present GVS method provides a more thorough modeling of the signal; thus, this method does not require the measurement location to be at the center of the labeled bolus. The GVS method also accounts for the blood spins that were extracted into tissue and then re‐exchanged into the blood. A drawback of the GVS method is that CBF information is needed in order to estimate the VTT and *E*, whereas the previous method can estimate *E* without needing CBF information. Our data suggested that the GVS method resulted in a lower inter‐session CoV compared to the peak‐detection method. We attribute this observation to two reasons. One is that there may exist day‐to‐day variations in VTT and, since the GVS method specifically accounts for VTT in its data fitting, the permeability results may be more reliable. The second reason is that the peak‐detection method relies on the identification of maximum signals across several spatial locations. Thus, noise may be amplified in the peak signal compared to the signal at a particular location.

Blood T_1_ is one of the major confounding factors that affect the WEPCAST signal intensity. In the human brain, the values range from 1550 to 2050 ms at 3 T, predominantly depending on the hematocrit (Hct) level and oxygenation saturation fraction [39, 40, 42, 49]. Our previous study showed that a 10% difference in blood T_1_ will result in > 10% errors (around ±15 mL/100 g/min) in PS [22], while the impact of brain disease on PS has been suggested to be approximately 20 mL/100 g/min [2, 8]. Therefore, the PS estimation may greatly benefit from acquiring an individual blood T_1_ in vivo. We implemented a saturation recovery sequence to measure blood T_1_ at the posterior SSS, using an approach similar to previous reports [39, 40, 41, 42]. Here, instead of using an inversion recovery sequence, we employed a saturation recovery sequence so that we do not need to use a long TR for the magnetization to recover and there are minimal spin history effects. During the acquisition, we used a Look‐Locker scheme, so that multiple delay times can be measured in the same TR. We applied a fixed interval between delay times so that the background signals are identical across time points, which makes it easy to correct during the model fitting. We found our venous blood T_1_ estimations to be reliable. Females generally have higher values than males, consistent with the sex differences in Hct [39, 50, 51]. We observed a clear benefit of spending an extra 1‐min measuring blood T_1_. Among healthy subjects, PS using individual blood T_1_ revealed a tighter distribution compared to using assumed blood T_1_, with an inter‐subject CoV of 6.8%. While there are expected physiological variations in PS across people, without considering individual T_1_, the measured PS may exhibit additional variance due to biases associated with the technique and/or model. Therefore, acquisition of blood T_1_ information may allow us to minimize the experiment‐related (i.e., artifactual) PS variations. Tight distribution of measurements among a normal group is critical for the sensitivity to detect a disease effect in patient populations.

The current study has some limitations. Although the GVS‐based method improves the reliability of WEPCAST MRI, the model requires additional assumptions, such as the standard deviation of VTT dispersion, a fixed ratio between $\delta_{a}$ and $\delta_{v}$, and tissue T_1_. Their effects on the estimation of PS are relatively small (as shown in Table 1), but nonetheless represent potential confounding factors. Because the present WEPCAST MRI sequence only provides two experimental measures (control and labeled signals), we are unable to estimate these parameters. Additional experimental data, for example, VTT distribution from VICTR MRI, may further improve the reliability of BBB permeability quantification. For blood T_1_, the present study has only measured it in a large vessel but has not accounted for potential variations across the venous vascular tree, especially in venules where Hct may be lower and T_1_ may be longer. Additionally, the present study has only enrolled young healthy volunteers. Although the utility of this method in middle‐aged participants has recently been demonstrated in a long‐COVID clinical study [9], the benefits of the GVS method in older participants need to be further tested.

## 5 Conclusion

The reliability of WEPCAST MRI for the measurement of BBB permeability can be enhanced using theoretical and experimental advancements. The data processing of WEPCAST MRI can be improved by applying a GVS‐model to both control and labeled signal intensities to simultaneously estimate water permeability and venous transit time. An extra measurement of blood T_1_ within 1 min was found to significantly reduce the inter‐subject variations in PS estimation. These technical advancements may allow WEPCAST MRI to be used as a robust tool for the assessment of BBB integrity in brain diseases.

## Data Availability

The data that support the findings of this study are available on request from the corresponding author. The data are not publicly available due to privacy or ethical restrictions.
